# Strategic implementation of transportation networks following the February 6th Kahramanmaraş Earthquake: an observational analysis

**DOI:** 10.1186/s13049-023-01129-2

**Published:** 2023-10-31

**Authors:** Sarper Yilmaz

**Affiliations:** Department of Emergency Medicine, University of Health Sciences, Kartal Dr. Lutfi Kirdar City Hospital and The secretary of the Disaster Commission of the Turkish Emergency Medicine Association, Kartal, Istanbul, Turkey

**Keywords:** Transport centers, Kahramanmaraş, Earthquake, Disaster Logistics, Disaster response, Mitigation plan

To the Editor,

Upon a thorough review of the letter to the editor titled “*Enhancing disaster response through comprehensive transportation models: insights from the Kahramanmaraş earthquakes*” penned by Tatliparmak and Ak, I felt compelled to contribute additional insights based on my observations, aligning with previous reports [1, 2]. While I am in agreement with the critiques offered by Tatliparmak and Ak, I wish to elaborate on specific nuances.

Historically, immediate neighboring settlements have always been the first line of aid during disasters. The events of February 6, 2023, exemplify this, as Kahramanmaraş’s Pazarcık and Elbistan districts in Turkey experienced twin earthquakes registering magnitudes of 7.8 Mw (± 0.1) and 7.5 Mw within a span of nine hours. In the subsequent period, more than 40,000 aftershocks, some with magnitudes as high as 6.7 Mw, were documented. The aftermath extended beyond a single province, affecting a staggering ten provinces and even bordering nations, encompassing a vast expanse of roughly 350,000 km^2^ and impacting approximately 16% of Turkey’s populace, translating to nearly 14 million individuals [3, 4].

In the wake of this calamity, ten provinces faced significant infrastructural damage, most notably the health facilities in Hatay, Kahramanmaraş, and Adıyaman, leading to a dire humanitarian crisis. With local healthcare systems compromised, many survivors sought care in neighboring provinces. Interestingly, certain medical institutions within the disaster’s epicenter persevered and offered services under formidable conditions. Immediate medical intervention became a paramount concern, necessitating rapid relocation to proximate, less affected medical establishments post-initial triage. The seasonal factors—given that the event transpired in February—compounded by the destruction, rendered several roadways inoperative, ushering the need for alternative transportation modalities beyond traditional ambulances.

Strategically, the Ministry of Health of the Republic of Turkey swiftly leveraged both civilian and military aerial assets. Initial evacuation operations prioritized moving patients from the disaster epicenters to key medical facilities situated in cities such as Diyarbakır, Mersin, and Adana, earmarked as primary transport hubs. After initial stabilization, inter-provincial transfers predominantly utilized aerial routes. Comprehensive data on the utilization of various aerial transport modes within the initial week post-disaster are presented [5]. Moreover, maritime avenues played a pivotal role, particularly given the crippled terrestrial infrastructure. Crucial supplies and rescue apparatus were dispatched to the affected zones, and the injured were evacuated via İskenderun port in Hatay [6]. Concurrently, vessels were repurposed to shelter the displaced, while the contribution of terrestrial transport is documented [7].

Land ambulances were instrumental in transporting casualties from the disaster’s epicenter to operational healthcare establishments. Following preliminary evaluations, severely injured individuals were shuttled to these primary hubs through air, sea, or land routes. Upon receiving primary interventions, they were subsequently relocated to tertiary care establishments in distant provinces. These hubs, thus, served as pivotal nodes in the emergency response mechanism, catering to trauma-induced medical exigencies [8–10].

The primary objective of this commentary is to underscore the pivotal role of aerial transportation and central hubs, particularly when urban centers confront significant disruptions to their healthcare infrastructure in the wake of catastrophic natural calamities or mass casualty events. Natural disasters can strain local medical resources, impeding their capacity to provide exhaustive medical interventions. The stratagem adopted by Turkey post the February 6th seismic events epitomizes the importance of prompt initiation of care during the initial stages of a disaster. Engaging in scholarly dialogues on this topic might prove invaluable for proactive disaster mitigation planning.

### Electronic supplementary material

Below is the link to the electronic supplementary material.

Supplementary Material 1

## Data Availability

Please contact author for data requests

## Declarations

### Meetings

The article has not been presented in any organization.
