# Supplementary material for: Strategic implementation of transportation networks following the February 6th Kahramanmaraş Earthquake: an observational analysis
Source: Scand J Trauma Resusc Emerg Med. 2023 Oct 31;31:66. doi: 10.1186/s13049-023-01129-2 (PMC10619220; doi:10.1186/s13049-023-01129-2)
Supplement: Supplementary file 1 — Supplementary Material 1 [file 13049_2023_1129_MOESM1_ESM.docx]

**7th October 2023**

**Cover letter**

Dear Editor,

I hope this letter finds you well. I am writing in response to your kind invitation to contribute to ***Scandinavian Journal of Trauma, Resuscitation and Emergency Medicine*** and would like to express my sincere gratitude for considering my participation.

The opportunity to contribute to Scandinavian Journal of Trauma, Resuscitation and Emergency Medicine is an honor, and I appreciate the chance to share my work with your esteemed readership. Upon a thorough review of the letter to the editor titled “Enhancing disaster response through comprehensive transportation models: insights from the Kahramanmaraş earthquakes" penned by Tatliparmak and Ak, I felt compelled to contribute additional insights based on my observations, aligning with previous reports. I expanded on my observations in my letter to the editor titled "Strategic Implementation of Transportation Networks Following the February 6th Kahramanmaraş Earthquake: An Observational Analysis".

I want to clarify that if any publication fees are associated with the acceptance of this manuscript, I would like to request a waiver. Unfortunately, due to budget constraints, I am unable to cover such fees at this time. Please let me know if this presents any challenges or if there are alternative arrangements that can be made.

I look forward to the possibility of contributing to ***Scandinavian Journal of Trauma, Resuscitation and Emergency Medicine*** and the opportunity to engage with the academic community through this publication. Please feel free to contact me if you require any further information or have any questions regarding my submission.

Thank you for your time, consideration, and understanding. I appreciate your dedication to advancing academic research and look forward to your response.

**Sarper Yılmaz, MD.**

Specialist of Emergency Medicine, M.D., Kartal Dr. Lütfi Kırdar City Hospital, Department of Emergency Medicine, Istanbul, Turkey**,** The secretary of the Disaster Commission of the Turkish Emergency Medicine Association, Ankara, TURKEY.

**Address:** Şemsi Denizer Cad. E-5 Karayolu Cevizli Mevkii 34890 Kartal / Istanbul

**E-Mail:** [sarperyilmaz08@gmail.com](mailto:sarperyilmaz08@gmail.com)

**ORCID ID:** 0000-0001-8166-659X

**Phone:** +90 05072801164
